# Blasto-ing through the chest wall: Empyema necessitans due to pulmonary blastomycosis

**DOI:** 10.1016/j.idcr.2025.e02185

**Published:** 2025-02-11

**Authors:** Cameron G. Gmehlin, Nicholas P. Bergeron, Vaishnavi Ramesh, Haris Akhtar, Zelalem Temesgen

**Affiliations:** a Department of Internal Medicine, Mayo Clinic, Rochester, MN, USA; b Division of Public Health, Infectious Diseases, and Occupational Medicine, Mayo Clinic, Rochester, MN, USA

**Keywords:** Blastomyces, Empyema Necessitans, Fungal, Pulmonary, Blastomycosis

## Abstract

Empyema necessitans is a rare complication of empyema and often associated with chronic, untreated infections. Here we report a case of a 28-year-old male who presented with cough, fever, pleuritic chest pain, and left chest wall mass who was found to have empyema necessitans caused by *Blastomycosis dermatiditis*. He was neither immunocompromised nor had exposures to soil or freshwater sources. Empyema necessitans caused by Blastomyces is rare, with only three other case reports published in the literature. We write to raise awareness of this phenomenon and highlight the importance of maintaining strong clinical suspicion for fungal etiologies of chronic constitutional and pulmonary symptoms, especially when unresponsive to empiric antibiotics.

## Introduction

Empyema, or superinfection of parapneumonic effusion, complicates approximately 2–3 % of all pneumonias [1]. Most often these infections are caused by pyogenic bacteria such as *Streptococcus pneumoniae, Staphylococcus aureus*, oral streptococci, and anaerobes [2]. Empyema necessitans is a rare complication of empyema, characterized by extension of purulent material through the chest wall to form a subcutaneous collection [3]. Rates of empyema necessitans have decreased since the advent of antibiotics as this complication is associated with chronic, untreated infections with most cases reported in the literature involving tuberculosis or actinomyces [4]. Blastomyces are a dimorphic fungus native to North America that is associated with exposure to contaminated soil and/or decaying organic matter [5]. Most commonly, exposure to Blastomyces results in pulmonary disease with a spectrum of clinical presentations from subclinical respiratory infections to fulminant acute respiratory distress syndrome [6]. We present an unusual case of empyema necessitans caused by *Blastomyces dermatiditis* in an immunocompetent patient that highlights the importance of considering fungal etiologies, even in patients without apparent risk factors or immunosuppression.

## Case

A 28-year-old male with no past medical history was admitted to our hospital for cough, fever, pleuritic chest pain, and a left chest wall mass. He initially presented to the Emergency Department six months prior for left anterior thoracic pain with cough. A chest x-ray revealed a left-midlung haziness but given the predominant symptom being left thoracic pain he was symptomatically treated for musculoskeletal pain. He was prescribed acetaminophen and ketorolac and discharged home. He subsequently developed a productive cough and worsening malaise, and he re-presented to his primary care provider one month later. Repeat chest x-ray demonstrated persistence of left lung findings, and he was treated for community acquired pneumonia with a 5-day course of amoxicillin. He initially experienced improvement of his cough and malaise, though subsequently sought evaluation at urgent care when these symptoms recrudesced. He was prescribed a 7-day course of amoxicillin/clavulanate and a 5-day course of prednisone. His symptoms never fully resolved, and one month later he noted a painful swelling over his left anterior chest wall. His primary care provider obtained a limited ultrasound which demonstrated a 1.3 cm x 6.0 cm x 4.4 cm fluid collection in the ventral chest wall. Subsequent CT abdomen/pelvis was obtained which demonstrated a consolidation in the left lower lobe of the lung associated with a loculated pleural effusion and soft tissue stranding extending through the chest wall with effacement of sixth rib and organized fluid collection in the chest wall consistent with an empyema necessitans (Fig. 2). He was subsequently directed to our hospital for further evaluation.

**Fig. 1:**
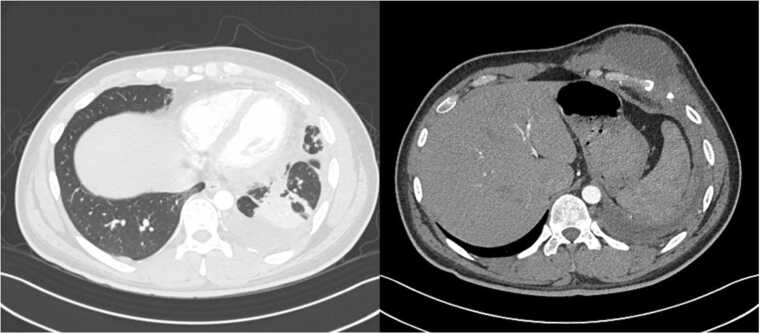
CT Chest/Abdomen/Pelvis demonstrating consolidation and atelectasis in the left lower lobe suspicious for pneumonia with an adjacent loculated pleural effusion concerning for empyema. Soft tissue stranding present through chest wall with effacement of left anterior sixth rib with additional organized fluid collection in lower chest consistent with empyema necessitans.

**Fig. 2:**
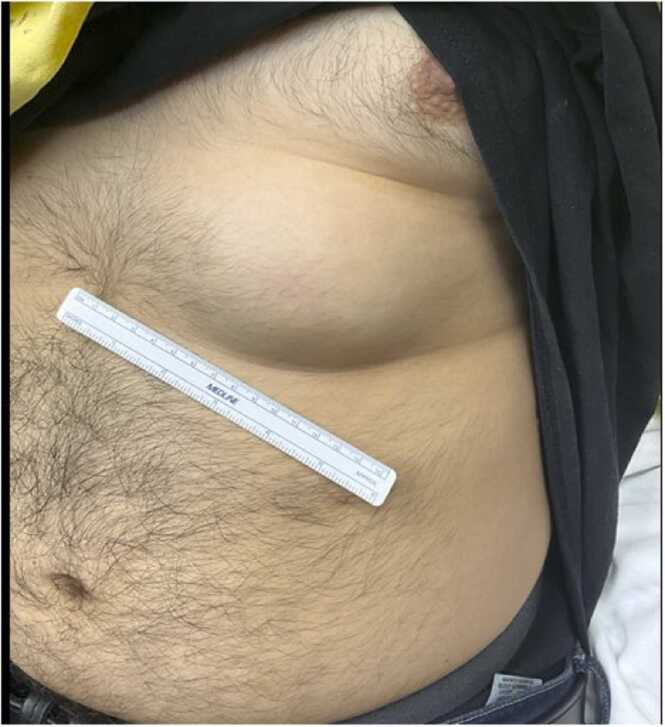
left chest wall mass appreciated during physical exam.

In the Emergency Department he was hemodynamically stable without deviation in vital signs. Laboratory testing was significant for a mild normocytic anemia, an elevated C-reactive protein of 46.0 mg/L (reference range <5.0 mg/L) and an elevated sedimentation rate of 56 mm/hr (reference range 0–22 mm/hr). Lung examination was notable for decreased breath sounds in the left lung base and crackles in the left middle/upper lung fields. Visual inspection revealed a palm-sized, subcutaneous nodule on the left anterior chest wall (Fig. 2). He was started on IV ceftriaxone, azithromycin, and metronidazole for suspected cavitary community acquired pneumonia and admitted for further management.

A multidisciplinary team was consulted including Infectious Diseases, Thoracic Surgery, Interventional Pulmonology, and Interventional Radiology. Further biographical discussion revealed that the patient was employed by a local grocery store and spent most of his time indoors. He denied any recent travel nor risk factors for tuberculosis. He owned a cat but had no other exposure to animals. He lived in an older domicile with an attic and basement that had a significant amount of water damage and had visible mold.

He underwent CT-guided placement of a chest tube into the left pleural space and a surgical drain into the left chest wall mass for diagnostic and therapeutic purposes. Results of the fluid analysis (Table 1) revealed two distinct fluid phenotypes. A broad array of microbiological testing on pleural fluid and serum was sent (Table 2). Sputum culture was ordered by was not obtainable due to inability to expectorate at that time. Bronchoscopy was not deemed necessary because patient was eupneic and saturating well on room air. Fungal smear of the pleural fluid revealed broad based budding yeast and subsequent combined serum Histoplasma/Blastomyces enzyme immunoassay (EIA) and fluid PCR was positive for *Blastomyces dermatitidis*. The patient was started on liposomal Amphotericin B (5 mg/kg IV daily) for 10 days of induction therapy with concurrent itraconazole loading (200 mg 3 times daily for 3 days). He was subsequently discharged with close Infectious Disease follow-up. Fungal cultures grew Blastomyces species complex confirming the diagnosis of Blastomycosis empyema necessitans. An outpatient sinogram 10 days after chest tube placement demonstrated complete resolution of his chest wall collection. Repeat serum Blastomyces antigen was undetectable. He developed refractory hypokalemia due to Amphotericin B and it was stopped after 10 days. He continued Itraconazole with plans to complete 1 year of therapy. At 2 month follow up, the patient had reported complete resolution of his fevers and shortness of breath. On physical examination, his left chest wall mass had resolved. His itraconazole level was 1.6 mcg/mL (ref range: >1 mcg/mL). Further imaging was deferred given his clinical improvement.

**Table 1:** Left pleural and chest wall abscess fluid characteristics.

Lab Value	Left Pleural Space	Chest Wall Abscess
Appearance	Serous	Slightly Bloody
pH	7.4	< 6.80
Total Cells (per mcl)	62	224,280
Neutrophils (%)	1	85
Lymphocytes (%)	89	0
Monocytes (%)	6	15
Eosinophils (%)	4	0
Lactate Dehydrogenase (U/L)	207	> 9000
Fluid Protein (g/dl)	4.9	5.7
Triglycerides (mg/dL)	18	0
Glucose (mg/dl)	63	2

**Table 2:** Microbiological evaluations.

Left Pleural Fluid Studies	
**Test**	**Result**
Gram Stain	Negative
Bacterial Culture	No growth after 5 days of incubation
Fungal Smear	Organisms resembling Blastomyces species complex
Fungal Culture	Blastomyces species complex
AFB Smear	Negative
MTB Complex PCR	Negative
Mycobacterial Culture	No growth after 42 days of incubation
Actinomyces Culture	No growth after 14 days of incubation
Nocardia Stain	Negative
Broad Range Bacterial PCR + Sequencing	Negative
Histoplasma/Blastomyces PCR	Positive for Blastomyces dermatitidis/gilchristii
*Other Serological Testing*	
**Test**	**Result**
Blood Cultures	No growth after 5 days
QuantiFERON-TB Gold Plus	Negative
HIV−1/−2 Ag and Ab Screen	Negative
Urine Legionella Antigen	Negative
Urine Streptococcus pneumoniae Antigen	Negative
MRSA nasal swab PCR	Negative

**Table 3:** Review of the literature.

Title	Author, Year, Journal	Presentation	CT Findings	Diagnostics	Treatment and Outcomes
A Rapidly Expanding Chest Wall Mass in an Adolescent With COVID−19	Klair et al., 2023, Clinical Pediatrics	15 y/o male with two days of mild, dry cough and expanding chest mass near left upper sternal border.	Dense consolidation appreciated in left upper lobe with extension into the pleural cavity and chest wall with associated intramuscular abscesses.	Grocott Methenamine Silver: budding yeast Periodic Acid-Schiff Stain: budding yeast Blastomyces Urine EIA: positive Blastomyces Serum EIA: positive Fungal Culture: Blastomyces DNA Sequencing: Blastomyces gilchristii	400 mg Itraconazole for 6 months with complete resolution of symptoms.
A Case of Pulmonary Blastomycosis Mimicking Pulmonary Tuberculosis	Byung Woo Jhun et al., 2012, Tuberculosis and Respiratory Diseases	45 y/o male with chronic cough. CT scan revealed right upper lobe lesion, which was biopsied, showing granulomatous inflammation. Patient was treated with anti-tuberculosis therapy with no improvement. Bacterial/Mycobacterial cultures were negative.	Mass-like infiltrative lesion in the right upper lobe with surrounding nodules. A well-defined low-density lesion in communication with subcutaneous tissue present near anterior right upper chest wall.	Gomori Methenamine Silver: budding yeast DNA Sequencing: Blastomyces dermatitidis	400 mg Itraconazole for 3 months with complete resolution of symptoms.
A Case of Chest Wall Blastomycosis	Matthew Nemoy et al., 2023, CHEST 2023 Annual Meeting Abstract	40 y/o male who presented to primary care clinic for persistent non-productive cough and dyspnea. Chest radiograph revealed left sided consolidation and associated pleural effusion and he was treated with amoxicillin/clavulanic acid without improvement. He subsequently was referred to the Emergency Department. A chest tube was placed and 1.5 liters of fluid was removed. Bacterial cultures and Cytology was negative.	Small residual effusion and lytic lesion of 9th rib. Repeat CT chest (4 weeks) showed new 4 × 7 cm mass along posterior left chest wall.	Needle Aspiration: granulomas, fungal forms Fungal Culture: Blastomyces dermatitidis	400 mg Itraconazole for 6 months with complete resolution of symptoms.

## Discussion

We present a rare case of empyema necessitans with rib osteomyelitis in which the causative organism was found to be *Blastomycosis dermatiditis*. While empyema necessitans is commonly caused by *Mycobacterium tuberculosis*, accounting for approximately 40 % of cases, other pathogens have been reported including Actinomyces species*, Streptococcus pneumoniae*, *Staphylococcus aureus*, and various gram-negative bacilli [7]. Fungal causes of empyema necessitans are rare but have been documented, including cases of aspergillosis and histoplasmosis [8], [9]. Blastomycosis has only been infrequently reported as the etiological agent of empyema [10]. During our review of the literature, there were only three other cases of empyema necessitans caused by Blastomycosis [11], [12], [13].

Unlike other fungal diseases such as histoplasmosis or coccidiomycosis, development of disseminated blastomycosis is not uncommon. Furthermore, it occurs at similar rates between immunocompetent and immunocompromised individuals [14]. In the case of our patient, he was classified as having moderate, disseminated blastomycosis. Thisemphasizes the need for clinicians to consider fungal etiologies even in patients who are without significant comorbidities or known immunosuppression. Another interesting aspect of this case is the absence of exposures traditionally associated with acquisition of pulmonary blastomycosis. Both *Blastomyces dermatitidis* and *Blastomyces gilchristii* are thought to be contracted through exposure to soil, specifically near lakes and rivers [5]. In the case of our patient, he denied outdoor activities, exposure to dust or dirt near lakes or rivers. Although the patient did not report classic risk factors for Blastomyces, studies indicate that a significant proportion (39 %) of individuals with Blastomycosis lack such identifiable exposures [14]. Residing in an endemic area (Mower County, MN) is likely the predominant risk factor for this patient’s infection [15].

Diagnosis of Blastomycosis is accomplished through antigen testing, direct visualization of fungal forms during histopathology or on fungal smear, molecular testing on specimen, or via culture. Our patient had all four modalities positive (antigen testing, fungal smear, fungal culture, and PCR). The gold standard for diagnosis remains fungal culture, though culture growth can take up to 1–4 weeks, leading to delays in diagnosis [5]. Identification is done by mass spectrometry (MALDI-TOF). If bodily fluid or tissue is readily available, diagnosis can often be rapidly accomplished if fungal forms are visualized on smear. In contrast to Candida and Aspergillus species, Blastomyces does not colonize or contaminate, meaning that detecting the organism on specimen or obtaining a positive culture is definitive for diagnosing blastomycosis. Histoplasma/Blastomyces serum antigen testing using EIA is a useful adjuvant testing modality which can be performed on either serum or urine. In a study of 67 patients with culture confirmed blastomycosis, the sensitivity of antigen testing was 82.7 % for urine and 64.3 % for serum in isolated pulmonary disease, while the sensitivity in disseminated disease was 66.7 % for urine and 33.4 % for serum in disseminated disease [16]. Because of significant cross-reactivity between the antigens of *Histoplasma* and *Blastomyces*, our center has combined these two antigen assays into a single test [17]. Finally, molecular testing using PCR is increasingly being utilized but lacks prospective evaluation studies for sensitivity and specificity. With the inherent limitations of current diagnostic modalities, diagnosis of blastomycosis is often delayed and can result in patients receiving multiple courses of ineffective antibiotics [18]. As in the case of our patient, delayed diagnosis can lead to serious complications including empyema necessitans.

The Infectious Disease Society of America has published guidelines on the treatment of Blastomycosis [19]. Treatment regimens are decided based upon whether Blastomycosis is pulmonary or disseminated and the clinical severity of disease. While there are no specific guidelines regarding treatment of empyema necessitans due to Blastomycosis, we elected to treat with a course of Amphotericin B upfront, followed by long term itraconazole based on recommendations for moderate disseminated extrapulmonary disease. Amphotericin has been shown to have good penetration into the pleural space and can effectively treat blastomycosis-associated pleural disease [20]. In another reported case of Blastomycosis empyema necessitans, itraconazole monotherapy was utilized with complete resolution of symptoms suggesting that it may be an acceptable alternative agent [11]. Total duration of therapy was set at 12 months given locally advanced disease and the presence of rib osteomyelitis. In our case, the patient’s 6th rib osteomyelitis is a significant complication. While bone involvement in blastomycosis is not uncommon and occurs in approximately 25 % of disseminated disease, the combination of empyema necessitans and rib osteomyelitis due to Blastomyces is exceptionally rare [6]. Finally, source control with chest wall drain placement and therapeutic thoracentesis lead to faster symptomatic relief and clinical stabilitzation.

In summary, our case highlights the importance of maintaining high clinical suspicion for endemic mycoses in patients with chronic constitutional and pulmonary symptoms who are unresponsive to antibiotics. Even without pathognomonic exposures or risk factors, patients may still be at risk for Blastomycosis infection in endemic regions of the world. Diagnosis of Blastomycosis often is delayed due to limitations of current diagnostic modalities, and severe complications requiring prolonged courses of antifungals such as empyema necessitans can occur. A multidisciplinary approach to treatment is required in disseminated disease.

## Ethical approval

Given the nature of this report IRB approval was waived.

## Consent

Patients included in this study have provided research authorization for the confidential clinical use of information to Mayo Clinic.

## Funding

None.

## CRediT authorship contribution statement

**Temesgen Zelalem:** Writing – review & editing, Conceptualization. **Akhtar Haris:** Writing – review & editing, Writing – original draft, Investigation, Conceptualization. **Ramesh Vaishnavi:** Writing – review & editing, Writing – original draft, Investigation. **Bergeron Nicholas P:** Writing – review & editing, Writing – original draft. **Gmehlin Cameron Gray:** Writing – review & editing, Writing – original draft, Investigation, Conceptualization.

## Declaration of Competing Interest

The authors declare the following financial interests/personal relationships which may be considered as potential competing interests: Editor-in-chief of IDCases: Z.T. If there are other authors, they declare that they have no known competing financial interests or personal relationships that could have appeared to influence the work reported in this paper.
